# *Ligilactobacillus salivarius* LZZAY01 accelerated autophagy and apoptosis in colon cancer cells and improved gut microbiota in CAC mice

**DOI:** 10.1128/spectrum.01861-24

**Published:** 2025-01-10

**Authors:** Wenhong Yang, Tao Li, Shixiang An, Rong Chen, Yuxin Zhao, Jiaxian Cui, Mingyu Zhang, Jingkun Lu, Yunpeng Tian, Lili Bao, Pengwei Zhao

**Affiliations:** 1 Laboratory of Microbiology and Immunology, School of Basic Medical Science, Inner Mongolia Medical University, Hohhot, China; 2 College of Animal Science and Technology, Tarim University, Alar, China; 3 Department of Anesthesiology, Inner Mongolia Chest Hospital (The Fourth Hospital), Hohhot, China; Ruhr-Universitat Bochum, Bochum, Germany

**Keywords:** *Ligilactobacillus salivarius*, LZZAY01, CAC, autophagy, apoptosis, tight junction protein, gut microbiota

## Abstract

Colorectal cancer (CRC) is one of the malignant tumors globally, with high morbidity and mortality rates. The mainstay treatment of CRC includes surgery, radiotherapy, and chemotherapy. However, these treatments are associated with a high recurrence rate, poor prognosis, and highly toxic side effects. The probiotics have the potential to prevent CRC, and they display a favorable safety performance. Probiotics could provide a potential strategy to prevent and treat CRC. The impact of LZZAY01 on cancer cell lines CT-26, HCT-116, and SW-620 was evaluated by conducting cytotoxicity and clonogenicity tests. A model of colitis-associated cancer (CAC) was established in C57BL/6j mice following induction with AOM/DSS. The levels of autophagy and apoptosis proteins, tight junction proteins, and inflammatory factors were detected by western blotting, immunofluorescence assay, and enzyme-linked immunosorbent assay. High-throughput sequencing of gut 16S rRNA was performed to analyze the abundance and diversity of the gut microbiome. LZZAY01, a new strain of *Ligilactobacillus salivarius*, was certified by an evolutionary tree and average nucleotide identity. LZZAY01 enhanced autophagy and apoptosis in CT-26, HCT-116, and SW-620 cell lines. It preserved the integrity of the intestinal barrier by regulating the tight junction protein ZO-1 and claudin-1. The tumor necrosis factor-α and interleukin-6 were reduced by LZZAY01. The abundance and diversity of the intestinal microbiota were enhanced, especially the beneficial bacterial species maintaining the balance of the intestinal flora such as *Bifidobacterium* and *Lactobacillus*. *L. salivarius* LZZAY01 improved CAC *via* suppressing the growth of colon cancer cells, promoting autophagy and apoptosis, enhancing intestinal tight junctions, reducing intestinal barrier degradation, modifying the gut microbiota abundance, and decreasing inflammatory reactions.

**IMPORTANCE:**

Although similar probiotics have been shown to have anticancer potential in colorectal cancer (CRC), there is a paucity of research related to the preventive function of probiotics against CRC. And there are fewer studies about the mechanism of probiotics’ preventive effects on CRC. The regulation of tumor cell proliferation and apoptosis by the active ingredients of probiotics may be one of the mechanisms of their prevention of CRC. In this study, we explored the effects of *L. salivarius* LZZAY01 on autophagy and apoptosis of colon cancer cells *in vitro* and *in vivo* and proposed a possible mechanism for the prevention of CRC by probiotics.

## INTRODUCTION

Colorectal cancer (CRC) is a prevalent malignant digestive tumor. According to the International Agency for Research on Cancer of the World Health Organization, CRC ranked third in incidence rate and second in mortality rate among malignant tumors globally in 2020 (1). CRC is influenced by hereditary, environmental factors, lifestyle, and dietary habits (2). The gut microbiota is intricately associated with CRC (3, 4).

Probiotics have several health-promoting properties. They have a clinical potential for treating diseases, such as irritable bowel syndrome, acute infectious diarrhea, and allergic diseases (5–7). Probiotics can be used as supplements to improve the functional properties of nutraceuticals and therapeutic preparations. There is a growing interest in developing probiotic adjuvants or alternative therapies for cancer. Certain probiotics, such as *Lactobacillus rhamnosus*, *Lactobacillus coryniformis*, *Lactobacillus salivarius*, and *Lactobacillus rhamnosus GG*, have demonstrated anticarcinogenic properties (8–11).

The intestinal mucosal barrier’s structural integrity is crucial for maintaining the body’s internal microenvironment and normal living activities. Tight junctions in intestinal epithelial cells are a crucial component of the intestinal mucosal structure (12), and dysregulation in the expression of tight junction proteins (TJPs) is a prognostic indicator for several diseases (13). TJPs largely consist of transmembrane proteins such as claudins and occludins, as well as the zonula occludens (ZO). The regulation of TJPs is associated with the advancement of colon cancer (14). Moreover, certain probiotics can regulate the expression of TJPs to repair the mucosal barrier by maintaining the microecological balance in the intestine and reducing intestinal inflammation (15).

In this study, we obtained LZZAY01, a novel strain of *Ligilactobacillus salivarius* to explore the function as new probiotics. We assessed the influence of probiotics on colon cancer cells and AOM/DSS-induced colitis-associated cancer (CAC) mice to explore possible mechanisms for CRC prevention.

## MATERIALS AND METHODS

### Gram staining of *Ligilactobacillus salivarius* LZZAY01

*Ligilactobacillus salivarius* LZZAY01 was incubated for 24 h in the MRS solution at 37°C with shaking for enrichment and afterward streaked onto the modified MRS solid medium (HuaiKai Microbial, China) with 0.8% CaCO_3_. Selected individual colonies were subjected to Gram staining and observed under a microscope.

### 16S rRNA sequencing to construct the genetic evolutionary tree of LZZAY01

The genome of *L. salivarius* was isolated using a bacterial whole-genome DNA kit. The universal primers used for amplifying bacterial 16S rRNA were 27F: 5′-AGAGTTTGATCCTGGGCTCAG-3′ and 1492R: 5′-CTACGGCTACCTTGTTACGA-3′. A 1,500 bp fragment size was achieved through PCR amplification and confirmed by visualizing the products on an agarose gel. The PCR products were retrieved using the AxyPrep DNA Gel Recovery Kit. The refined PCR results underwent DNA sequencing with an ABI3730-XL sequencer. The sequence was examined for similarity in the GenBank database to determine the bacterial species and construct a genetic tree.

### Whole-genome sequencing

Libraries were constructed using a whole-genome shotgun method, where different insert fragments were examined using second-generation sequencing techniques and third-generation single-molecule sequencing technologies on the Illumina NovaSeq and PacBio Sequel platforms. The Illumina TruSeq Nano DNA LT library preparation protocol was followed to create the necessary genomic onboard libraries using the TruSeq DNA Sample Prep Kit. The standard Pacbio Template Prep Kit 1.0 was utilized for third-generation single-molecule sequencing to create the necessary genomic onboard libraries. The unpaired loops of the libraries were joined to the sequencing primers and polymerase through annealing. Next, the binding samples were secured in zero-mode waveguide wells for sequencing.

### Genome assembly and analysis

Second-generation sequencing data underwent quality control using FastQC. High-quality sequences were produced by filtering the original downstream data. The third-generation downstream data were assembled using Unicycler and Flye software to obtain contig sequences. High-quality data from the second iteration were refined using the Pilon program on the third iteration contig outcomes, resulting in the completion of the full-length sequence. The effect of the assembled complete sequence was evaluated, and the assembled plasmid sequence was compared with the plasmid database. Furthermore, the genome sequence was cross-referenced with the nucleotide sequence (NT) database to obtain the species information of the genome.

### Gene prediction and annotation

Protein-coding genes were predicted from whole-genome sequences using the GeneMarks software. Genome-wide tRNA genes were identified using tRNAscan-SE, whereas rRNA genes were predicted using Barrnap. The remaining ncRNA predictions were obtained largely by comparing them with the Rfam database. Pathogenic bacterial virulence factor (VFDB) analysis, antibiotic resistance (CARD) analysis, phage prediction, gene island prediction, carbohydrate-activating enzyme (CAZy) analysis, and pathogenic bacterial bioattack factor annotation and MvirDB were conducted to identify bacterial subsystems. Subcellular localization analyses of genes encoding proteins included signal peptide prediction, transmembrane helices prediction, secretory protein prediction, secretion system protein and T3SS effector protein prediction, and secondary metabolite gene cluster analysis. Functional annotation of coding protein genes was conducted using NR, eggNOG, KEGG, Swiss-Prot, GO annotations, and TCDB, Pfam, PHI, and MEROPS database annotations.

### Comparative genome analysis

The whole-genome sequence information of *L. salivarius* LZZAY01 was compared with those of *L. salivarius* LPM01, ZLp4b, IBB3154, S32, ZLS006, DJ-sa-01, BNS11, JCM1046, 2102-15, AR809, CICC 23174 VHProbi A17, P1CEA3, SNK-6, 2D, UCC118, Ren, CECT 5713, and L. S.05 for average nucleotide identity (ANI) to assess their affinity at the strain level. The ANI values were computed after blast comparisons of multiple genomes using the OrthoANI software.

### Cell culture

Cultures of mouse CT-26, human HCT-116, and SW-620 colon cancer cells, along with human colon epithelial cell NCM460 (all the cell lines were purchased from ORiCells Biotechnology, China), were maintained in Dulbecco's Modified Eagle's Medium (DMEM) (Gibco, China) with 10% fetal bovine serum, penicillin-streptomycin (100 IU/mL penicillin, 100 µg/mL streptomycin) (Gibco, China).

### Preparation of bacterial culture with cell-free supernatant

The LZZAY01 strain was cultivated as single colonies in *Lactobacillus* MRS Broth with constant shaking at 220 rpm and 37°C for 12 h. The conditioned medium was collected and filtered through a 0.22 µm filter, once the bacterial density reached 10^9^ CFU/mL. The cell-free supernatant (CFS) was stored at −80°C for future use.

### Acid resistance and bile salt tolerance

The viability of the isolates in an acidic environment was determined according to the method described in the study by Zhang et al. (16) and Kuppusamy et al. (17) (16, 17). Acid tolerance was assessed using MRS broth (pH 5.0, pH 4.0, and pH 3.0), and bile tolerance was tested using MRS broth supplemented with 0.1%, 0.2%, and 0.3% bile salts. Then, 2,700 µL of each solution was mixed with 300 µL of each overnight culture in 5 mL tubes and incubated at 37°C for 4 h. The mixture was retrieved by pipette and measured at optical density 600. Survival was calculated as follows: strain survival rate = A1/A0 × 100%.

Where A0 denotes for the optical density at 0 h and A1 stands the optical density at 4 h.

### Adhesion of LZZAY01 to the NCM460 epithelial colon cell line

Adhesion experiments were performed following the method described by Kushwaha and Muriana, with slight modifications (18). The ability of NCM460 epithelial colon cells to adhere to the intestinal wall has been described previously. NCM460 colon epithelial cells were seeded into six-well plates at a concentration of 5 × 10^5^ cells per well. The cells were cultured until an 80% confluent monolayer was formed. Individual colonies were selected in the MRS culture and incubated at 37°C on a shaker set at a continuous speed of 220 rpm for 12 h until reaching the late logarithmic phase with around 10^9^ CFU/mL of LZZAY01. The number of cells in a single well of the plate was counted, and organisms were cultivated alongside NCM460 cells for 2 h at 37°C based on the MOI (100:1). The suspension was prepared by digesting and diluting it serially with phosphate-buffered saline (PBS). Later, 100 µL of the thinned solution was spread on the MRS solid medium and incubated for 48 h at 37°C. The amount of LZZAY01 attached to NCM460 cells was measured as CFU/well. The average number of LZZAY01 in each well was divided by the total number of NCM460 cells to calculate the LZZAY01 adherence rate. The experiment was repeated thrice, and three parallel images were computed each time.

### Cell viability assay

Cell viability for CT-26, HCT-116, and SW-620 cells was determined by conducting the CCK8 test at a starting cell count of 5,000 cells per well. Different levels of CFS were applied to the cells for 12, 24, and 48 h. Subsequently, the cells were treated with the CCK8 solution (Biosharp, China) for 1 h. The absorbance was recorded at 450 nm utilizing an enzyme marker (Thermo, China).

### Cell clone formation assay

A total of 700 logarithmic growth phase cells were seeded per well in six-well plates. After the CFS treatment, the plates were incubated for 7 to 14 days. Midway through the incubation period, the cells were examined, and the solution was changed every 2 to 3 days. The experiment was concluded once the number of clonal cells in each well exceeded 50 cells. The cell solution was rinsed with PBS before being removed. Afterward, the cells were exposed to 4% paraformaldehyde for 30 min, and stained with crystal violet for 20 min, followed by acquiring images and measuring the results.

### Inducing AOM/DSS for constructing a CAC model

Twenty male C57BL/6j mice [specific pathogen free (SPF) grade, purchased from Beijing Vital River Laboratory Animal Technology, China], aged 8 weeks, were randomly divided into four groups (*n* = 5/group): a normal control group (saline only), an AOM/DSS group (AOM/DSS only), a high-dose LZZAY01/H group (AOM/DSS and LZZAY01 1 × 10^10^ CFU/kg/day), and a low-dose LZZAY01/L group (AOM/DSS and LZZAY01 1 × 10^8^ CFU/kg/day). Colon cancer development was initiated by injecting 10 mg/kg of AOM (Sigma, Germany) intraperitoneally on the first day (19). After consuming 2.5% DSS (Yeasen Biotechnology, China) in their drinking water daily for 1 week, the subjects were subsequently switched back to normal drinking water for the following 2 weeks. The cycle was repeated thrice (20).

### Histopathological analysis

The tissue from the far end of the colon, measuring around 1 cm, was kept in a 4% paraformaldehyde solution at 4°C overnight. After being embedded in paraffin, the specimen was sliced into 5 µm sections for hematoxylin and eosin (H&E) (Solarbio, China) staining. A Leica microscope (LEICADM4000) was used to examine the stained sections and acquire images.

### Immunoblotting

The radioimmunoprecipitation (RIPA) (Beyotime Biotechnology, China) lysate was prepared by lysing tissue/cells on ice for 30 min, followed by sonication to extract the total proteins. Equivalent amounts of proteins were isolated by SDS-PAGE, followed by transfer onto nitrocellulose membranes and examination using primary antibodies. The membranes were treated with antibodies against Beclin-1, LC3II/I, BAX, BCL-2, and GAPDH (Cell Signal Technology, United States of America) and detected using an enhanced chemiluminescence reagent.

### Immunofluorescence

The tissue samples were deparaffinized, subjected to antigen retrieval, and then exposed to 0.1% Triton X-100 for 5 min. The solution of goat serum was slowly added drop by drop over the course of 1 h. After the exclusion of the inclusion solution, primary antibodies against ZO-1, Claudin-1, and Ki67 (1:400, Proteintech, China) were added and incubated overnight at 4°C. Following the washing of the primary antibodies with PBS, red and green fluorescent markers were applied sequentially.

### Enzyme-linked immunosorbent assay

Interleukin (IL)-6 and tumor necrosis factor (TNF)-α were detected in mouse serum according to the instructions provided on the IL-6 and TNF-α Micro Enzyme-Linked Immunosorbent Assay Kit (MEIBIAO Biology, China).

### High-capacity sequencing of 16S rRNA in the gut microbiome

Twenty fecal samples were obtained from each of the control, AOM/DSS, and LZZAY01L/H groups, with five samples from each animal. The Magnetic Soil/Stool DNA Kit (TIANGEN, China) was used to extract the whole-genome DNA from the samples, following the manufacturer’s instructions. The PCR products were sequenced using the Illumina NovaSeq technology. Clustering was conducted using the QIIME2 procedure, and every eliminated sequence produced by DADA2 noise reduction was labeled as an amplicon sequence variant.

### Statistical analysis

Statistical analysis was conducted using the GraphPad Prism 8.0 software (GraphPad Software, USA). The data are presented as mean ± standard error. An independent *t*-test was conducted on normally distributed data with chi-squared variance to compare data between two groups. One-way analysis of variance was utilized to compare data among several groups, and the difference was deemed significant with a two-tailed *P* value less than 0.05.

## RESULTS

### *L. salivarius* LZZAY01 genomic characteristics and comparative

The LZZAY01 displayed a smooth, moist surface, and clean edge on the MRS broth (Fig. S1A). Gram staining (Fig. S1B) was positive, with short rods and no spores. It was preliminary identified as a *Lactobacillus* spp.

**Fig 1:**
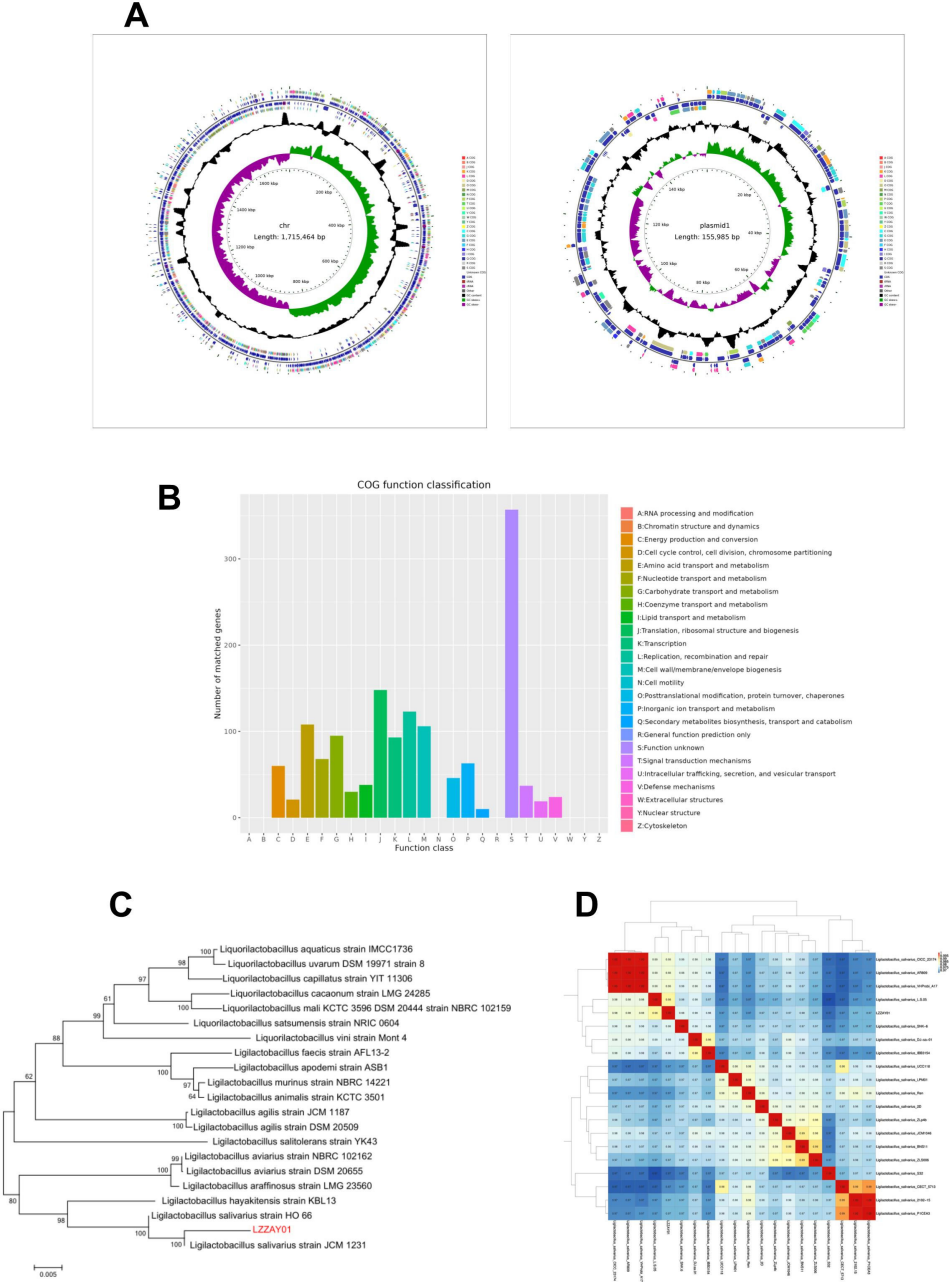
Genetic characteristics and phylogenetic analysis of LZZAY01. (**A**) Gene circle diagram of chromosome and plasmid: from inside to outside, first circle represents scale, second circle represents GCSkew, third circle represents GC content, fourth and seventh circles represent the COG to which each CDS belongs, and fifth and sixth circles represent the position of CDS, tRNA, and rRNA on the genome. (**B**) Functional classification diagram of bacterial eggNOG (COG) (Chr). (**C**) Phylogenetic tree. (**D**) ANI and phylogenetic analysis.

The genomic characteristics of *L. salivarius* LZZAY01 were demonstrated by the second generation with the third-generation contigs (Fig. 1A). The total yield of circular chromosomes was 1,715,464 bp (GC contents of 33.02%) and that of circular plasmid 1 was 155,985 bp (GC contents of 32.37%) (the gene sequence has been uploaded to the NCBI, GenBank accession number for nucleotide sequence: >SUB14446212 Z3277-10 PP790711). In total, 1,745 proteins in the open-reading frame (ORF) of the LZZAY01 genome, including 161 protein genes in the plasmid sequence, were identified by GeneMarkS. In addition, 135 non-coding RNAs (22 rRNAs, 77 tRNAs, and 36 ncRNAs) were identified (Table 1). COGs were divided into 25 categories (Fig. 1B).

**TABLE 1:** Characteristics of the *L. salivarius* LZZAY01

Attributes	Values
Genome size (bp)	1,715,464
GC content (%)	33
No. of sequences	1 chromosome (1 plasmid)
ORFnum	1,584
ORF total length (bp)	1,501,152
5S rRNA	8
165 rRNA	7
23S rRNA	7
tRNA	77
ncRNA	36
CRISPRs	7

The 16SrRNA gene sequence analysis revealed that the *L. salivarius* LZZAY01 strain had more than 99% sequence similarity to other *L. salivarius* strains. The LZZAY01 strain was tentatively coined *L. salivarius* based on the phylogenetic analysis (Fig. 1C). The whole-genome sequence information of *L. salivarius* LZZAY01 was different from other *L. salivarius* with whole-genome information in GenBank database by ANI analysis (Fig. 1D). The *L. salivarius* L.S.05 strain was intricately associated with the highest mean ANI value of 0.98. These data suggested that LZZAY01 was a new *L. salivarius* strain.

### Characteristics of LZZAY01

**Fig 2:**
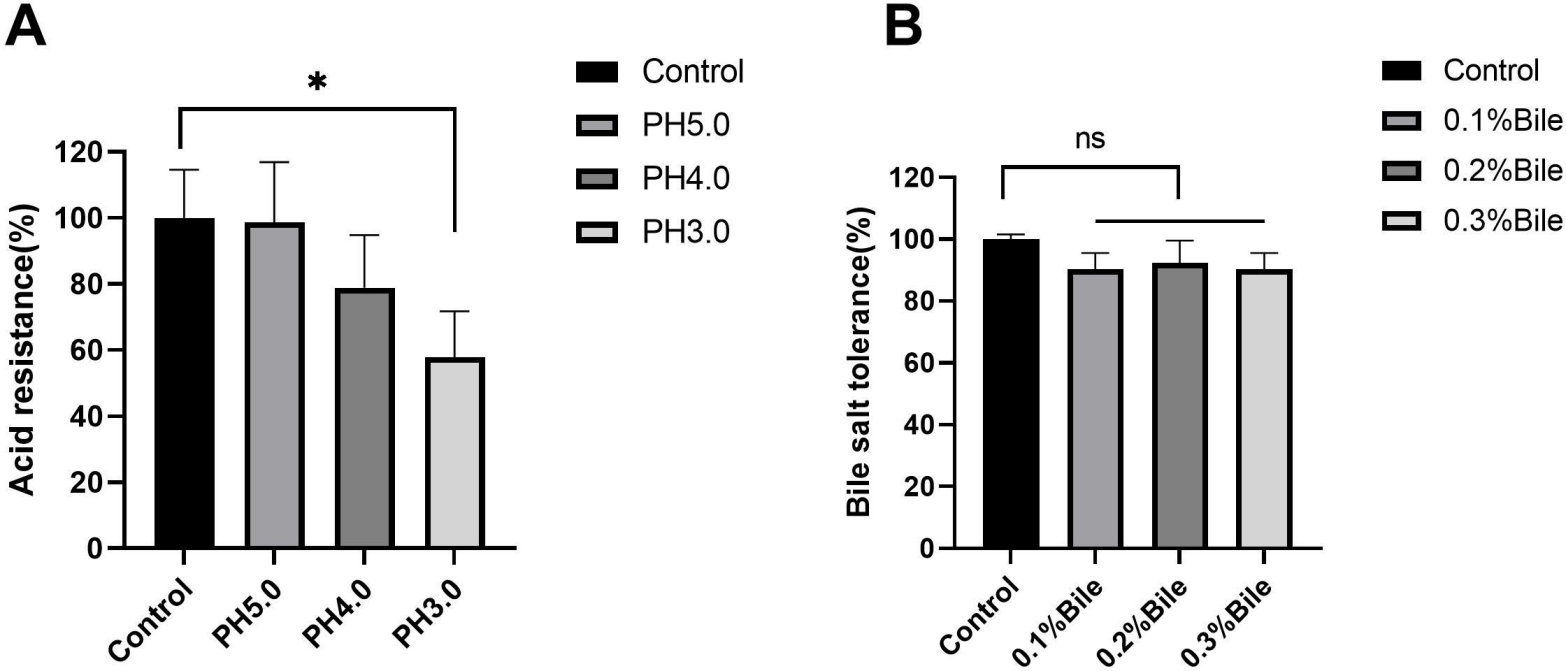
Assessment of acid and bile salt resistance of LZZAY01. (**A**) Bile salt resistance of LZZAY01 strain (ns, no statistically significant difference compared with control). (**B**) Survival of LZZAY01 in MRS medium of different pH (*P < 0.05, compared with control).

The LZZAY01 had significant acid tolerance (Fig. 2A). No significant difference was recorded at pH 5.0 and 4.0 compared with the control group. However, at pH 3.0, more than 50% of bacteria survived (*P* < 0.05). The bacteria exhibited high resistance to bile salt. The survival rate in the MRS medium with 0.1% to 0.3% porcine bile salt was not significantly different from the control group (Fig. 2B).

To demonstrate adhesion, the LAAZY01 strain was co-cultured with human intestinal epithelial cell line NCM460. We found that 11.58 ± 1.79 LZZAY01 could adhere to one intestine cell. The probiotic adhesion to the intestine cell was another key element to probiotic survival and plantation. This contributed to the survival and plantation of LZZAY01 in the intestine.

### LZZAY01 suppressed the proliferation and colony formation of colon cancer cells

**Fig 3:**
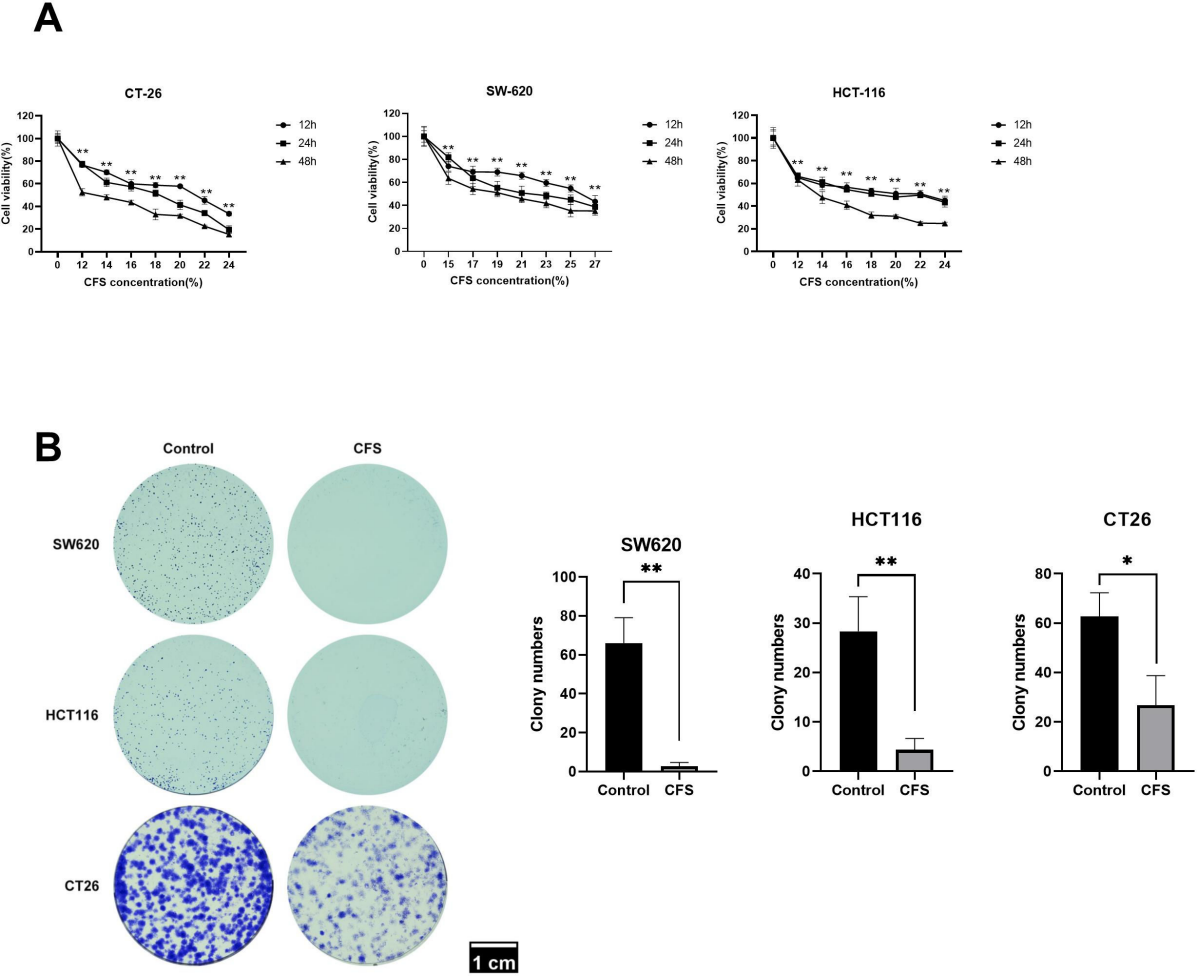
Inhibition of colon cancer cell activity by LZZAY01 CFS. (**A**) Mouse colon cancer CT-26 cells and human colon cancer cells HCT-116 and SW-620 cell line CCK8 assay (**P < 0.01, compared with control). (**B**) *In vitro* clone formation assay of three colon cancer cells. Control, control group; CFS, LZZAY01 strain CFS-treated group (*P < 0.05 and **P < 0.01, compared with control group). HCT-116, human colon cancer HCT-116 cell line; SW-620, human colon cancer SW-620 cell line; CT-26, mouse colon cancer CT-26 cell line.

The CFS of LZZAY01 inhibited the proliferation of CT-26, HCT-116, and SW-620 cell lines (Fig. 3A). The proliferation of CT-26, HCT-116, and SW-620 cells was related to the concentration and time of CFS. We detected the cell copy number to study the colony formation ability of CFS on colon cancer cells. The CFS suppressed the colony numbers in CT-26, HCT-116, and SW-620 cells (Fig. 3B). Altogether, the CFS of LZZAY01 suppressed the proliferation and colony formation ability of colon cancer cells.

### LZZAY01 induced autophagy and apoptosis in colon cancer cells

**Fig 4:**
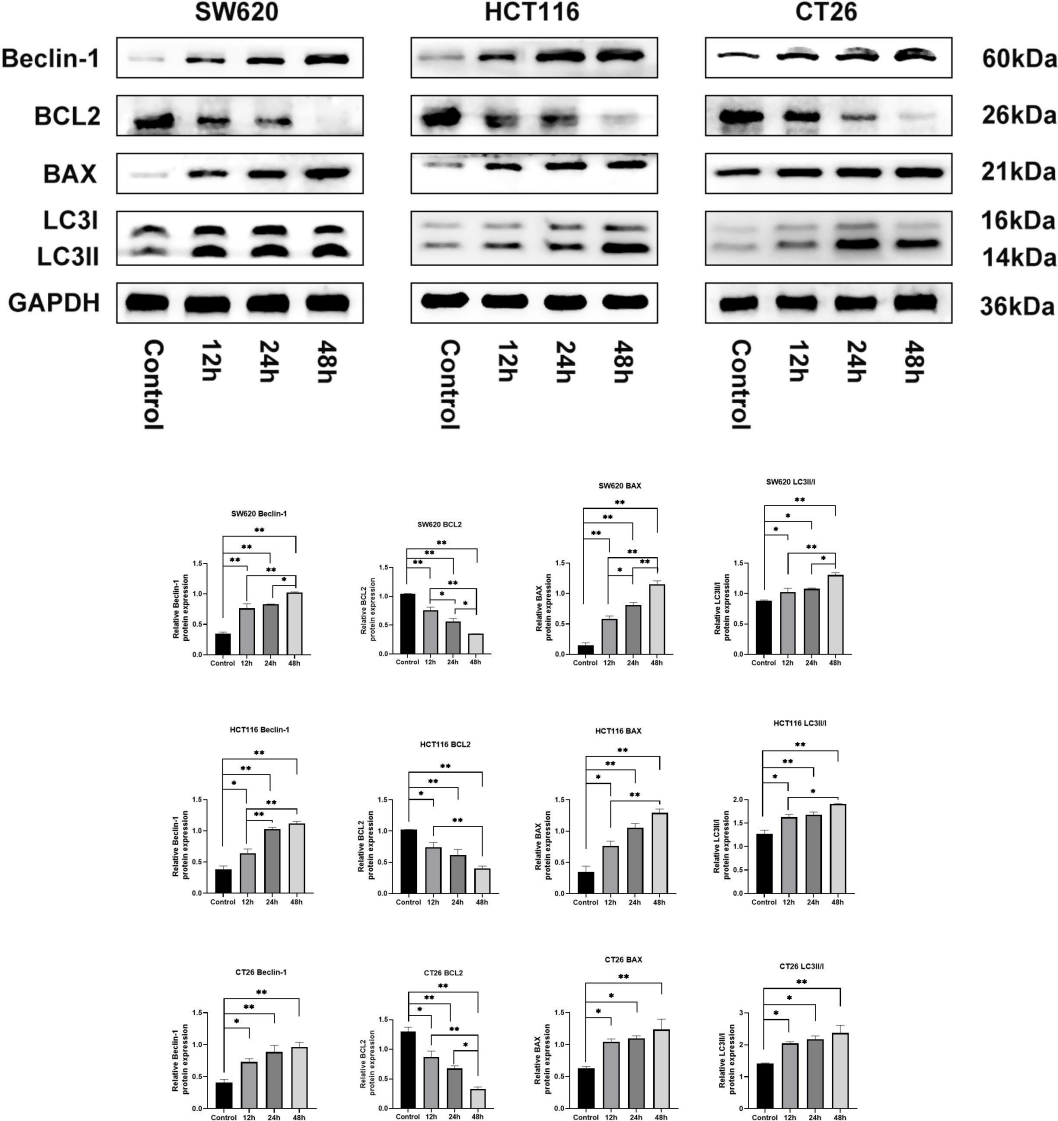
LZZAY01 promotes Beclin-1, LC3II/I, and BAX protein expression and inhibits BCL-2. Beclin-1, LC3II, LC3I, BAX, and BCL-2 western blot. Control, control group; 12 h, 24 h, and 48 h: CFS-treated group with each time (*P < 0.05 and **P < 0.01, compared with control group).

We next investigated the role of CFS of LZZAY01 in autophagy and apoptosis, for which the autophagy proteins Beclin-1 and LC3II/I and the apoptosis proteins BCL2 and BAX were determined in CT-26, HCT-116, and SW-620 cell lines. The expression of Beclin-1 and LC3II/I after 12 h, 24 h, and 48 h of CFS treatment was higher than that in the control group (Fig. 4). The level of BCL2 after 12 h, 24 h, and 48 h of CFS treatment decreased, whereas that of BAX increased (*P* < 0.05). Altogether, these data show that LZZAY01 induced autophagy and apoptosis in colon cancer cells.

### LZZAY01 delayed the initiation and advancement in CAC mice

**Fig 5:**
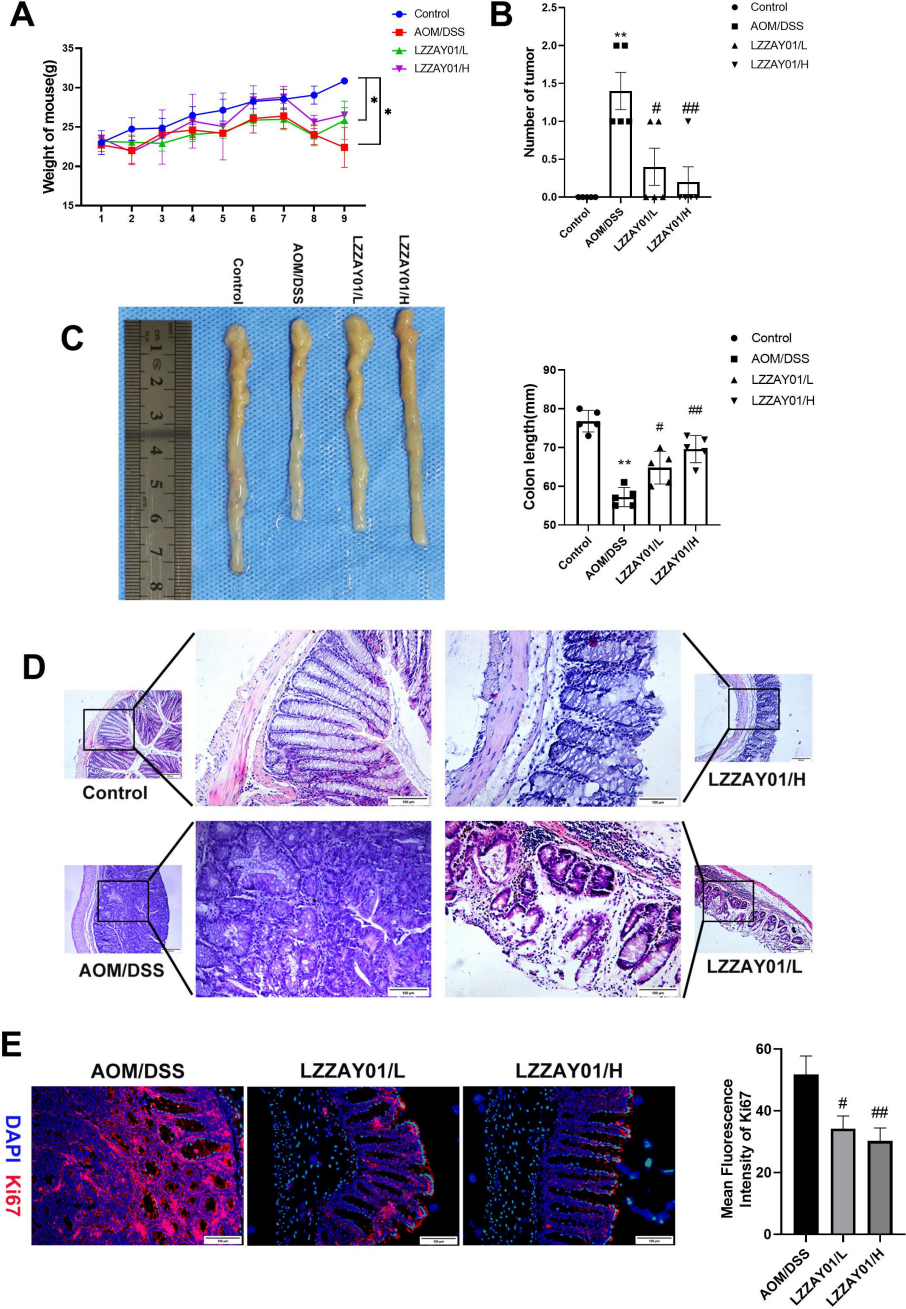
LZZAY01 delays tumorigenesis and progression in AOM/DSS-induced mice *in vivo*. (**A**) Change in body weight of mice in each group. Control, normal control group; AOM/DSS, AOM/DSS control group; LZZAY01/L/H, model groups of AOM/DSS treatment with simultaneous oral administration of low and high doses of LZZAY01, respectively (*P < 0.05, compared with normal control group); (**B**) number of tumors in the colon of mice in each group (**P < 0.01, compared with normal control group; #*P* < 0.05 and ##*P* < 0.01, compared and AOM/DSS group); (**C**) length of mouse colon in each group (*P < 0.01, compared with normal control group; #*P* < 0.05 and ##*P* < 0.01, compared with AOM/DSS group); (**D**) H&E staining of mouse colon tissue of each group (100× and 200× magnification); (**E**) Ki-67 immunofluorescence staining (scale bar = 100 µm).

We established CAC mice via induction with AOM/DSS to determine the anti-colon cancer function of LZZAY01. The high-dose LZZAY01 could recover mouse weight (Fig. 5A). The tumor number was reduced following treatment with LZZAY01 (*P* < 0.05), especially with the high-dose LZZAY01 (Fig. 5B). The colon length in the AOM/DSS group was notably shorter compared with that in the control group (*P* < 0.05). The colon length in the LZZAY01 group was shorter than that in the control group; however, it was longer than that in the AOM/DSS group (Fig. 5C). The LZZAY01 strain, especially its high dose, reduced the damage in colon tissues (Fig. 5D). The expression of Ki67 was diminished by high-dose LZZAY01 (Fig. 5E). These results showed that high-dose LZZAY01 effectively delayed the progression of CRC in CAC mice.

### LZZAY01 induced colon tissue autophagy and apoptosis in CAC mice

The role of LZZAY01 on autophagy and apoptosis in colon tissues was next studied, for which the expression of Beclin-1, LC3II/I, and t BCL2, BAX was determined. The expression of Beclin-1 and LC3II/I in the high-dose LZZAY01 group was increased compared with that in the AOM/DSS group (*P* < 0.05). In the AOM/DSS group, the level of BCL-2 in the high-dose LZZAY01 group was lower and that of BAX was higher than in the AOM/DSS group (Fig. 5A). These results suggested that high-dose LZZAY01 promoted autophagy and apoptosis in CAC mice.

### LZZAY01 improved intestinal barrier function in CAC mice

**Fig 6:**
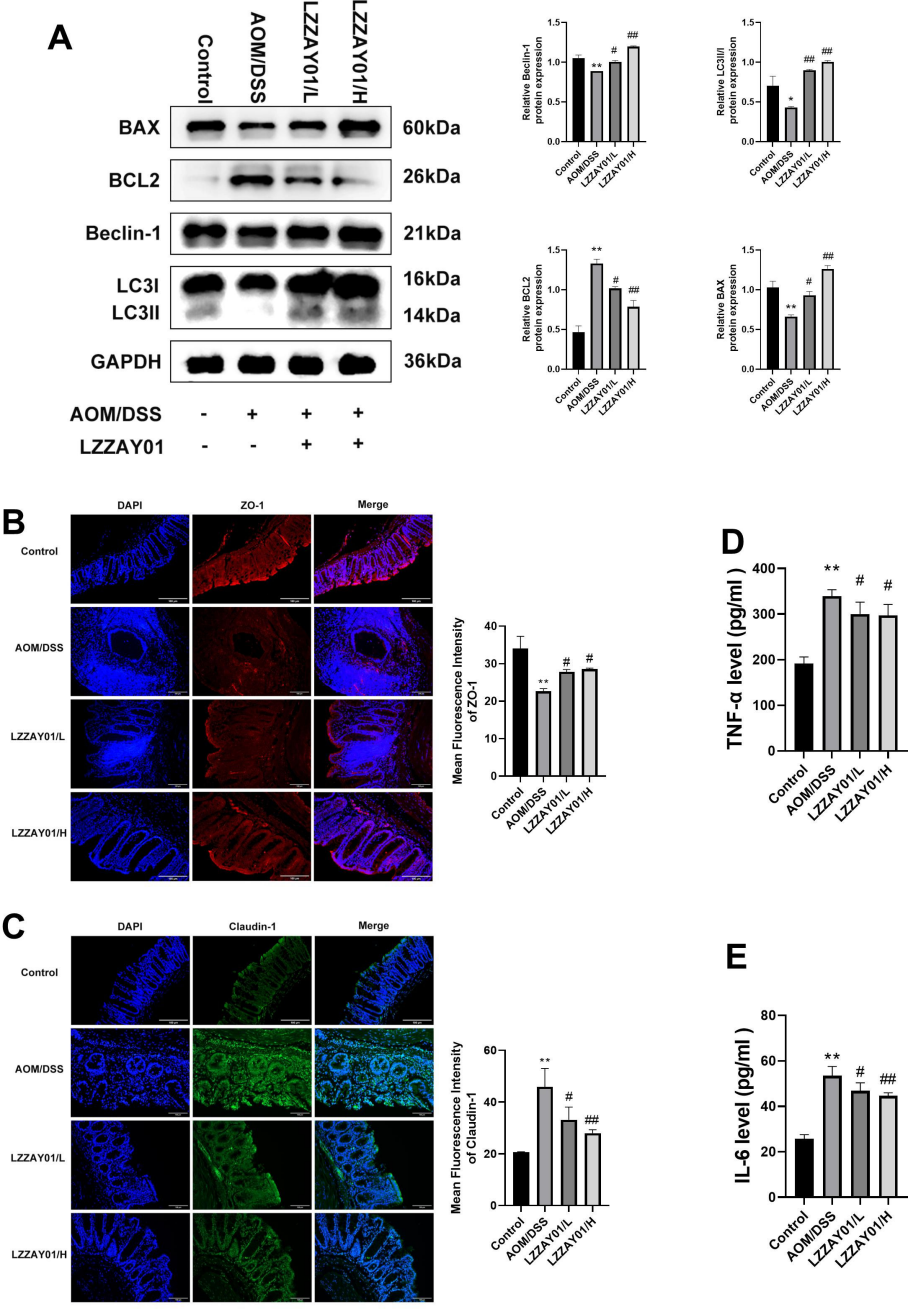
The expression levels of Beclin-1, LC3II/I, BCL-2, BAX, ZO-1, and Claudin-1 proteins, as well as the inflammatory factors TNF-α and IL-6, in each group of mice. (**A**) Expression of Beclin-1 and LC3II/I, BCL-2, and BAX in the colon tissues of mice in each group (western blot); (**B, C**) expression of TJPs ZO-1 and Claudin-1 in the colon tissues of mice in each group (tissue immunofluorescence); (**D, E**) inflammatory factors TNF-α and IL-6 in each group of mice *in vivo* expression levels (**P < 0.01, compared with normal control group; #*P* < 0.05 and ##*P* < 0.01, compared with AOM/DSS group).

Intestinal barrier function plays a crucial role in CRC. Hence, we examined the expression of intestinal TJPs in mice colon tissues. Compared with the control group, the expression of ZO-1 was decreased in the AOM/DSS group. Treatment with LZZAY01 recovered the expression of ZO-1 (Fig. 6B). The expression of Claudin-1 was also detected. LZZAY01, especially high-dose LZZAY01, declined the expression of Claudin-1, which was activated by AOM/DSS (Fig. 6C). Altogether, these data showed that LZZAY01 upregulated ZO-1 expression and downregulated that of Claudin-1 in the colon tissue in CAC mice. LZZAY01 treatment improved the intestinal barrier function in CAC mice.

### LZZAY01 decreased TNF-α and IL-6 in CAC mice

We next studied the inflammatory reaction by detecting the levels of TNF-α and IL-6. In the AOM/DSS group, the levels of TNF-α and IL-6 were significantly elevated, compared with those in the control group (*P* < 0.05). However, in the high-dose LZZAY01 and low-dose LZZAY01 groups, the levels of TNF-α and IL-6 were lower than those in the AOM/DSS group (Fig. 6D and E). Collectively, these findings showed that LZZAY01 decreased inflammatory reactions in CAC mice.

### LZZAY01 alleviated microbial dysbiosis and rebuilt gut microbiota in CAC mice

**Fig 7:**
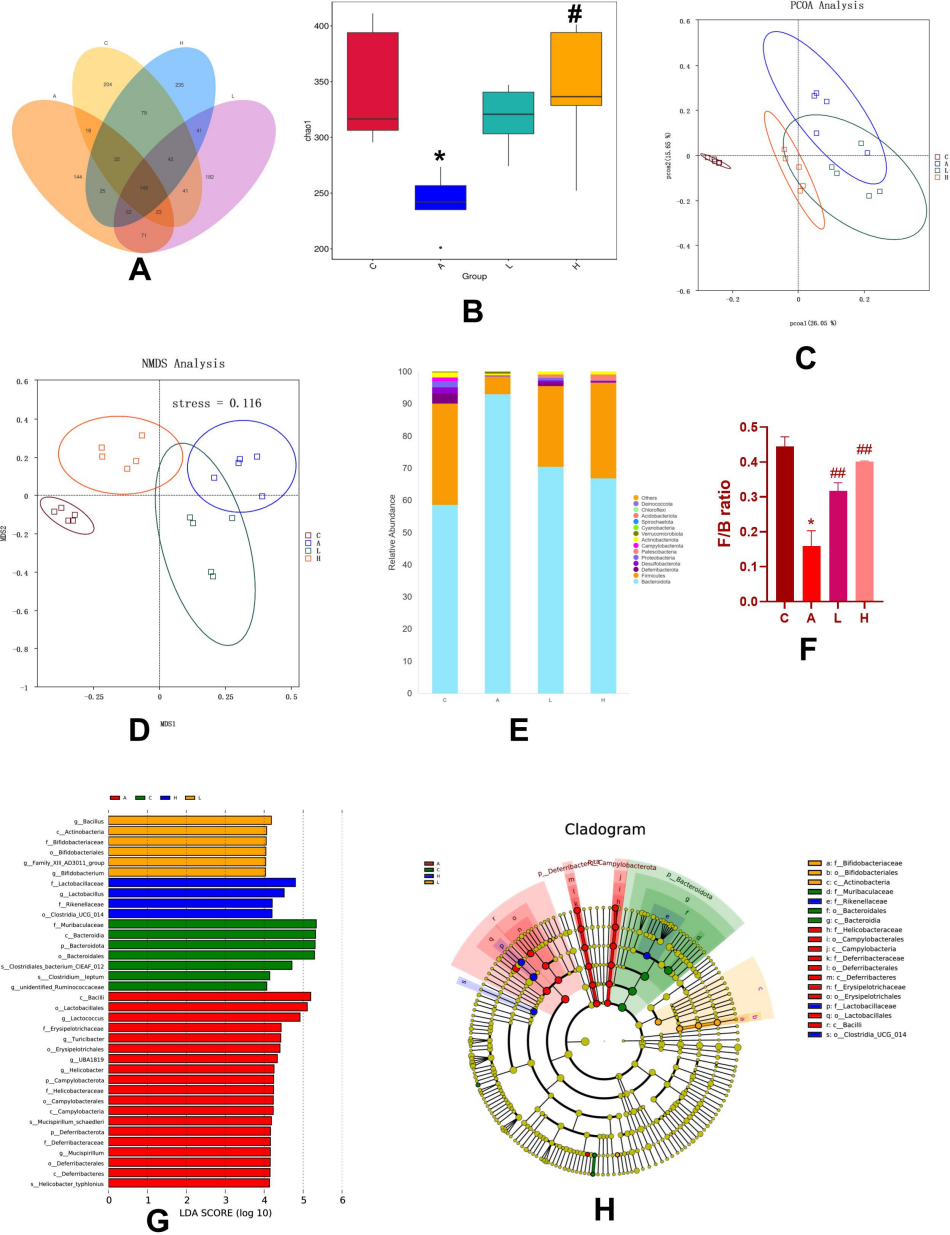
Analysis of the intestinal flora of mice by 16SrRNA sequencing. (**A**) Venn diagram of the number of AVS in the intestinal flora of each group. (C, control group; A, AOM/DSS group; L, LZZAY01/L group; H, LZZAY01/H group). (**B**) Chao1 index analysis (*P < 0.05, compared with the control group; #*P* < 0.05, compared with the AOM/DSS group). (**C**) PcoA analysis. (**D**) NMDS analysis. (**E, F**) Histogram of relative abundance of species (**P < 0.05, compared with the control group; ##*P* < 0.01, compared with the AOM/DSS group). (**G, H**) LefSe analysis.

Microbial dysbiosis often occurs in CRC. The species abundance in the AOM/DSS group was significantly lower than that in the control group. The number of OTUs in high-dose LZZAY01 and low-dose LZZAY01 groups was higher than that in the AOM/DSS group (Fig. 7A). The Chao1 index analysis plot illustrated the differences in community richness among groups, with a higher Chao1 index indicating more community richness. In the high-dose LZZAY01 group, the expression of Chao1 was significantly higher than that in the AOM/DSS group (Fig. 7B). The control and AOM/DSS groups were located in distinct quadrants by principal component analysis (PCoA) and non-metric multidimensional scaling (NMDS). The species makeup of the colonies in the high-dose LZZAY01 group was more similar to that in the control group (Fig. 7C and D). The distinct gut microbiota of mice in each group mostly included *Bacteroides*, thick-walled *Bacteroides*, *Actinobacteria*, *Micrococcus*, *Campylobacter*, *Aspergillus*, and *Desulfovibrio* (Fig. 7E and F). Compared with the control group, the AOM/DSS group decreased the abundance of *Firmicutes* and increased that of *Bacteroidota*. The *Firmicutes/Bacteroidota* (F/B) ratio was decreased. LZZAY01 treatment recovered Firmicutes and Bacteroidota, balancing the F/B ratio. LefSe analysis showed a decrease in the abundance of beneficial bacteria such as *Bifidobacterium* and *Lactobacillus*, in the AOM/DSS group, whereas an increase was observed in LZZAY01 groups (Fig. 7G and H). Altogether, LZZAY01 alleviated microbial dysbiosis and rebuilt the gut microbiota in CAC mice.

## DISCUSSION

CRC is a highly malignant cancer with a high incidence and mortality rate (1, 21). Treatments for CRC are associated with high recurrence rates, strong toxicity, side effects, and poor prognosis (22, 23). Probiotics, as food-derived substances, are safe and suitable for long-term use and could prevent CRC (24, 25). Certain probiotics can suppress the progression of CRC in preclinical experiments (26–29). The mechanisms to therapeutic cancer, especially with respect to CRC, have remained elusive. Several researches have focused on the probiotics obtained from humans such as feces and intestine (8, 10). In this study, LZZAY01 was isolated and purified from the saliva of black chickens on the edge of the Taklamakan Desert, which is an extremely arid area. The LZZAY01 was confirmed as a new strain by the ANI analysis. The LZZAY01 may have a role in CRC treatment. We found that LZZAY01 improved CAC by suppressing the proliferation of colon cancer cells, promoting autophagy and apoptosis. LZZAY01 enhanced the intestinal barrier function by regulating the expression of ZO-1 and Claudin-1. LZZAY01 regulated the balance of intestinal flora and reduced the release of pro-inflammatory factors IL-6 and TNF-α.

The survival of probiotics in the digestive system such as the stomach and intestinal tract has been a major challenge (30). Hence, probiotics can resist the damage by acids in the stomach and bile salts in the intestinal tract. In this study, we found that LZZAY01 could resist acid (pH 3.0) and 0.3% bile. LZZAY01 could reach the intestine through the stomach avoiding damage by acids and bile. The probiotic adhesion to the intestine cells was another key element to probiotic survival and plantation (16, 31). We found that 11.58 ± 1.79 LZZAY01 could adhere to one intestine cell. Hence, LZZAY01 could live in the intestinal tract.

In this study, the CRC-suppressive function of LZZAY01 was demonstrated. LZZAY01 suppressed the proliferation of colon cancer cells, promoting autophagy and apoptosis *in vivo*. We built an *in vitro* CAC model in mice by inducing with AOM/DSS. LZZAY01 retarded the tumor number and promoted autophagy and apoptosis. Excessive autophagy leads to autophagic cell death and subsequent cancer cell elimination (32, 33). In addition, promoting apoptosis inhibits colon cancer progression (34, 35).

The intestine barrier function was damaged in the CAC model by AOM/DSS-induced mice (36, 37). In this study, the LZZAY01 could repair intestinal damage by inhibiting the expression of claudin-1 and promoting ZO-1. Claudin-1 and ZO-1 belonged to the TJP family. The main function of TJPs is to protect intestinal barrier function from damage by bacteria and viruses (38, 39). Especially in CRC, Claudin-1 and ZO-1 play a key role in preserving the intestine barrier function (40–42). The levels of inflammatory cytokines were also disrupted in CAC model mice. IL-6 and TNF-α were elevated in CAC mice, as previously studied (43, 44). We found that LZZAY01 treatment declined the expression of IL-6 and TNF-α in CAC mice.

The occurrence and progression of CRC are closely related to gut microbiota (4, 45). The gut microbiota was destructed in CAC mice (46, 47). The LZZAY01 strain recovered the gut microbiota. LZZAY01 enhanced the F/B ratio and abundance of *Bifidobacterium* and *Lactobacillus*. The Firmicutes-to-Bacteroidota (F/B) ratio is often used to assess colon inflammation, with a decrease in the F/B ratio, indicating disease activity (48, 49). The role of *Bifidobacterium* and *Lactobacillus* in gastrointestinal disorders is established (50, 51). Increased abundance of *Bifidobacterium* and *Lactobacillus* can prevent and assist in the treatment of colon cancer (52, 53).

### Conclusions

*L. salivarius* LZZAY01 improved CAC by suppressing the growth of colon cancer cells, promoting autophagy and apoptosis, regulating intestinal tight junctions, reducing intestinal barrier degradation, modifying the gut microbiota, and decreasing inflammatory reactions.

## Data Availability

All data generated or analyzed during this study are included in this published article and supplied materials. LZZAY01 gene data were submitted in NCBI with accession number CP158282-CP158283.

## SUPPLEMENTAL MATERIAL

Figure S1 spectrum.01861-24-s0001.docx Colony morphology and Gram staining of Ligilactobacillus salivarius LZZAY01.

ASM does not own the copyrights to Supplemental Material that may be linked to, or accessed through, an article. The authors have granted ASM a non-exclusive, world-wide license to publish the Supplemental Material files. Please contact the corresponding author directly for reuse.
