# Supplementary material for: Ligilactobacillus salivarius LZZAY01 accelerated autophagy and apoptosis in colon cancer cells and improved gut microbiota in CAC mice
Source: Microbiol Spectr. 2025 Jan 10;13(2):e01861-24. doi: 10.1128/spectrum.01861-24 (PMC11792455; doi:10.1128/spectrum.01861-24)
Supplement: Figure S1 — Colony morphology and Gram staining of Ligilactobacillus salivarius LZZAY01. [file spectrum.01861-24-s0001.docx]

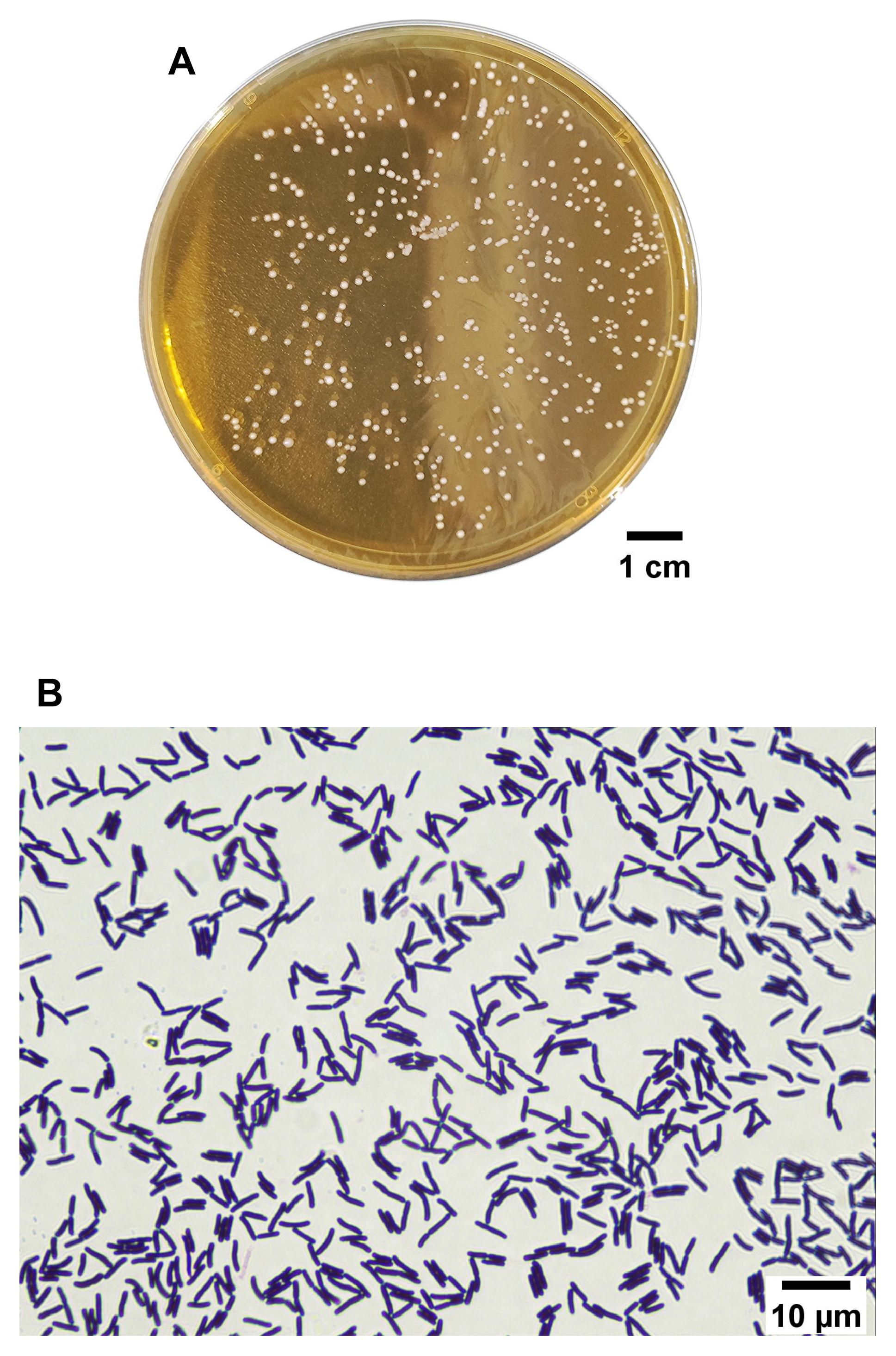


supplemental Figure1 Colony morphology and Gram staining of Ligilactobacillus salivarius LZZAY01. (A) LZZAY01 colonies grown in MRS medium. (B) Gram staining.
